# Removing Iron and Organic Substances from Water over the Course of Its Treatment with the Application of Average and Highly Alkaline Polyaluminium Chlorides

**DOI:** 10.3390/molecules26051367

**Published:** 2021-03-04

**Authors:** Izabela Krupińska

**Affiliations:** Faculty of Civil Engineering, Architecture and Environmental Engineering, Institute of Environmental Engineering, University of Zielona Góra, 15 Prof. Z. Szafrana St, 65-516 Zielona Góra, Poland; i.krupinska@iis.uz.zgora.pl; Tel.: +48-68-3282560

**Keywords:** water treatment plants, organic substances, iron compounds, iron-organic complexes, average and highly alkaline polyaluminium chlorides, highly alkaline polyaluminium chlorides modified with iron

## Abstract

In topic-related literature pertaining to the treatment of water, there is a lack of information on the influence of iron ions in highly basic polyaluminum chlorides on the efficiency of purifying water with increased contents of organic substance. The aim of this work was to determine the changes in the content of organic substances as well as iron compounds in water intended for human consumption following unit treatment processes with particular attention paid to the coagulation process. As coagulants, polyaluminium chloride PAXXL10 with an alkalinity of 70%, as well as polyaluminium chloride PAXXL1911 with an alkalinity of 85% the composition of which also contained iron, were tested. The analysis of the obtained results showed that iron compounds and organic substances were removed to the greatest extent by the coagulation process, which also had a significant influence on the final efficiency of water treatment. The effectiveness of water treatment was determined by the type of tested polyaluminum chloride, which influenced the formation of iron-organic complexes. The reason behind the formation of colored iron-organic complexes during coagulation using PAXXL1911 coagulant was the high pH (approx. 8), at which the functional groups of organic substances, due to their dissociation, are more reactive in relation to iron, and possibly the fact of introducing additional iron ions along with the coagulant.

## 1. Introduction

The necessity of removing organic substances from water is essential, ideally in the first processes of its treatment, seeing as how organic substances are the precursors of oxidation and disinfection by-products, and in the case of groundwater—also make it difficult to remove iron compounds, forming colored and stable iron-organic complexes with iron (II) and iron (III) [1,2,3,4].

Depending on the type and content of organic substances, as well as the pH and the oxidation-reduction potential, iron compounds, along with organic ligands, may form iron-organic complexes characterized by various degrees of solubility, which are difficult to remove during the conventional technological groundwater treatment system [5,6,7].

According to many authors [8,9,10,11], one of the reasons behind iron being stabilized by organic substances in groundwater may be the formation of so-called protective colloids of a hydrophilic nature, which are created as a result of the adsorption of organic substances on the surface of iron (III) hydroxide. It is also possible that, in natural waters, iron may create mixed complexes with organic or non-organic ligands. It was moreover confirmed that the constant durability of the iron-organic complex increases along with an increase in pH, which is most likely caused by the growing dissociation of functional groups –COOH and –OH, as well as the lower competitiveness of H^+^ ions when compared to metals, which occurs in an acidic environment [5].

Organic substances found in purified water are also precursors of by-products of oxidation. Most often, during the disinfection of water, but also in the water supply network, organic substances reacting with chloride as a result of a substitution reaction, form chloroorganic compounds characterized by cancerogenic and mutagenic properties. Aldehydes (e.g., formaldehyde)—the products of oxidizing organic substances with ozone—are also dangerous, revealing very high mutagenic activity [12,13,14,15].

Coagulation and filtration traditionally applied in order to remove organic substances are oftentimes unsuccessful. It is possible to intensify the removal of organic substances from water during coagulation through the application of optimal parameters of the process, including the type and dose of coagulant as well as the optimal pH, time, and mixing intensity. Coagulation, which aims to efficiently remove the precursors of disinfection by-products is referred to as intensified coagulation. The efficiency of the coagulation process significantly influences the final of water purification in the entire technological system.

In order to intensify the coagulation process, pre-hydrolyzed coagulants such as polyaluminium chlorides, are increasingly frequently applied in systems for purifying water intended for human consumption [16,17].

Polyaluminium chlorides, as trade products, are characterized by alkalinity ranging from 15 to 90%. Individual types of polyaluminium chlorides vary in terms of alkalinity (low-, average-, and highly-alkaline), aluminum content, chloride ion content, the presence or lack of modifiers (cation modifiers, anion modifiers), the so-called mass module, that is the ratio of aluminum content to chlorides, as well as the degree of polymerization of the compound, that is the structure of the composition of particles, the types of their bonds, and the distribution of the surface charge. Coagulants of low alkalinity are characterized by alkalinity below 40%, average alkalinity (from 40 to 70%) and highly alkaline (from 80 to 85%).

Thanks to pre-hydrolysis relying on the partial neutralization of aluminum chloride under strictly controlled conditions (temperature, pressure), polyaluminium chlorides are characterized by increased alkalinity, which, in consequence, results in the natural alkalinity of water being used up to a lesser degree as well as not lowering the pH as much when compared to aluminum or iron sulfates (VI), at the same time leading to the lower intensification of the corrosiveness of water following coagulation [18,19,20].

In solutions of polyaluminium chlorides, there are more polymerized forms with a high positive charge than among the products of the hydrolysis of coagulants that had not been subjected to pre-hydrolysis. The amount of monomers and polymers in polyaluminium chlorides varies and depends mainly on the value of the alkalinity coefficient of coagulants (r = [OH^−^]/[Al^3+^]).

Along with the increase in the alkalinity of polyaluminium chlorides (r) from 1.0 to 2.0, the dominant form is a polymer [Al_13_O_4_(OH)_24_]^7+^ called the “Al_13_” polymer, whereas for r > 2.1, the share of Al_13_ polymers begins to decrease, since they are precipitated in aluminum hydroxide. The maximum share of Al_13_ is indicated for an alkalinity of approx. 70 to 80%, which corresponds to an r value of 2.1 [19,21]. A further increase in the alkalinity of the coagulant leads to a decreased share of these forms, that is lower effectiveness of coagulation carried out based on the mechanism of neutralizing the charge [22,23,24,25]. It was shown that the degree of removing DOC depends on the content of Al_13._ The efficiency of lowering UV_254_ is, on the other hand, determined by the presence of Al_13_ and precipitated forms, whereas the decrease in water turbidity is influenced mainly by the forms of precipitated aluminum. Various modifiers in small amounts can be added to polyaluminium chlorides in small amounts of up to 5%, e.g., sulfates (VI) aiding the precipitation of aluminum hydroxide and the sedimentation of post-coagulation suspensions or silicate increasing the effectiveness of flocculation and the sedimentation of floccules [26]. Calcium, on the other hand, can be added in order to reduce the corrosion potential of purified water as well as the dose of coagulant required to remove total organic carbon [27]. Polyaluminium chlorides can also be modified with iron ions, which, over recent years, have appeared on the Polish market [28]. In topic-related literature pertaining to the treatment of drinking water, there is a lack of information on the influence of iron ions in highly basic polyaluminium chlorides on the efficiency of purifying water with increased contents of organic substance.

The aim of this work was to determine whether the presence of iron ions in highly basic polyaluminium chlorides and increasing the pH of the water during the coagulation process influences the final efficiency of purifying water obtained in the entire technological process due to the possibility of creating additional colloidal or dissolved iron-organic complexes between organic substances present in the water and iron ions introduced along with the coagulant. The use of high-base polyaluminium chlorides may facilitate the formation of iron-organic complexes due to increasing the water pH to ≥8 during the coagulation process, which could cause complete dissociation of organic substances and this creates good conditions for the formation of iron-organic complexes [4,5,6,7,8,16,29,30]. The research was conducted on a laboratory and technical scale. During the research of studies carried out on a laboratory scale, the efficiency of the coagulation process was carried out with the use of the jar test. As coagulants, the commercial average and highly alkaline polyaluminium chlorides were tested. PAXXL10 with an alkalinity of 70% and alkalinity coefficient of r = 2.10, as well as PAXXL1911 polyaluminium chloride with an alkalinity of 85% and alkalinity coefficient of r = 2.55, which, in its composition, also contained iron in the amount of approx. 0.7%. The research on a technical scale was carried out at a water treatment plant "Zawada" in Poland. The subject of the study was a mixture of surface water from the Obrzyca River and the groundwater after the aeration process from Quaternary formations. 

## 2. Results and Discussion

### 2.1. Water Samples

The characteristics of the physical and chemical composition of groundwater, groundwater after aeration, and surface water from the Obrzyca River prior to and after the microfiltration process, as well as mixed water, are presented in Table 1.

**Figure 1:**
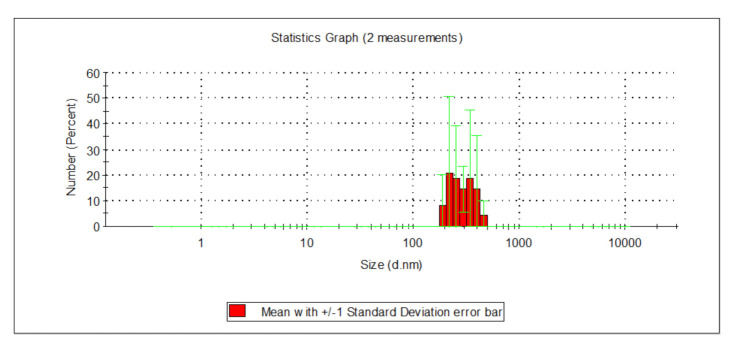
Distribution of particle sizes in mixed water.

The collected surface water, as well as groundwater, were characterized by increased contents of organic substances (Table 1). Total organic carbon content in groundwater and surface water was: 6.000 and 10.110 mg_C_/L respectively, whereas the content of dissolved organic carbon was 5.800 and 9.431 mg_C_/L, respectively. Organic substances containing aromatic rings were present among the dissolved organic substances, both in the groundwater as well as the surface water. In the case of groundwater, absorbance in UV_254_ and UV_272_ was 14.49 and 12.94 m^−1^, and in the case of surface water, 23.110 and 19.060 m^−1^. The color at wavelengths of 340 and 410 nm for groundwater were 20 and 23 mg_Pt_/L, whereas, in the case of surface water, these values were 36 and 48 mg_Pt_/L. According to the literature [31,32], water containing natural organic substances with chromophoric groups reveals an absorbance in the range of 200 to 340 nm. This indicates that active aromatic rings containing oxygen-rich functional carbonylic, carboxylic, and hydroxylic functional groups, as well as alkenes chains with double bonds are present in their structure. Color measured at a wavelength of 340 nm indirectly characterizing the value of colourful organic substances [33]. The groundwater was characterized by excessive contents of iron at 2.353 mg_Fe_/L and manganese at 0.240 mg_Mn_/L, as well as a high turbidity of 15 NTU. In such water, iron (II) and iron (III) compounds were present (Table 1). The value of the electrokinetic potential (ζ) for groundwater was −14.3 mV, and in the case of surface water −10.47 mV. The analyzed groundwater and surface water samples also differed in terms of the value of the coefficient of the co-presence of organic substances and total iron, calculated as D = (TOC)/(Iron_total_) (g_C_/g_Fe_) and D’ = (DOC)/(Iron_total_) (g_C_/g_Fe_). The value of the D and D’ coefficient for groundwater was: 2.549 and 2.465, and 78.984 and 73.679 in the case of surface water, whereas the value of the SUV_254_ indicator for groundwater was 3.144 m^2^/g_C_ and 2.450 m^2^/g_C_ for surface water. SUVA_254_ indicator values in the range of 2–4 m^2^/g_C_ signify that both hydrophilic, as well as hydrophobic and also small- and large-particle natural organic compounds, are present in the water [8,16,31]. The particle diameter present in the groundwater was in the range from 295 to 1484 nm, and in surface water from 142 to 396 nm. Mixed water (groundwater after the aeration process and surface water following microfiltration process at a volumetric ratio of 1:2) was characterized by an increased total iron content of 1.080 mg_Fe_/L and increased turbidity of 15 NTU, with the intensity of color at wavelengths of 340 and 410 nm being 26 and 40 mg_Pt_/L respectively, a pH of 8.10 and electrokinetic potential (ζ) −11.4 mV. TOC reached values of 8.231 mg_C_/L, DOC 7.827 mg_C_/L, and UV_254_ and UV_272_ absorption—19.338 and 16.165 m^−1^, which indicates that organic substances containing aromatic rings, which are characterized by a high potential of forming oxidation or disinfection by-products, were present among the dissolved substances in the purified water. The value of the D and D’ potential in mixed water was 7.621 and 7.247, with a calculated SUVA_254_ value of 2.471 m^2^/g_C_, which in turn signifies that both hydrophilic as well as hydrophobic substances, as well as small and large-particle organic compounds, were present in the water, with a dominance of non-humic, hydrophilic substances of low molecular weights. The measurement of the size of particles using a Zetasizer Nano analyzer showed that the particles present in the mixed water measured from 190 to 459 nm (Figure 1).

### 2.2. Removing Iron and Organic Substances from Water over the Course of Its Treatment

Based on the obtained research results, the effectiveness of removing organic substances, as well as iron compounds from water in individual processes of the technological system was determined. The efficiency of the following processes was assessed: aeration, microfiltration, coagulation, filtration through a catalytic-oxidative sand bed covered with iron and manganese oxides, filtration through a dolomite filter bed, and disinfection with chlorine dioxide.

#### 2.2.1. Aeration

The analysis of the obtained research results presented in Table 1 showed that, despite an increased concentration of organic substances, the aeration of groundwater in cascades with forced airflow led to an approx. 95% effectiveness of oxidizing iron compounds (II), an increase in turbidity from 15 to 18 NTU, as well as color measured at a wavelength of 410 nm, from 23 to 34 mg_Pt_/L, and a slight increase in color measured at a wavelength of 340 nm, from 20 to 22 mg_Pt_/L. The aeration of groundwater also caused an increase in pH from 7.31 to 7.70, as well as an increase in the average particle size, i.e., from 848 to 2473 nm.

#### 2.2.2. Microfiltration

**Figure 2:**
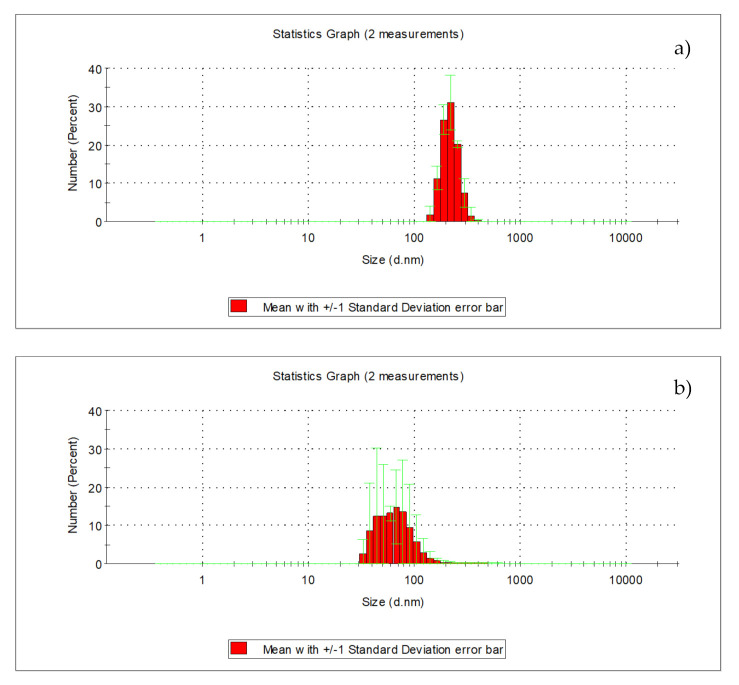
Distribution of particle sizes in surface water before microfilters (**a**) and following microfilters (**b**).

The microfiltration process on drum microfilters with pores measuring 10 µm caused turbidity to reduce from 2.10 to 1.40 NTU, and the total iron concentration from 0.128 to 0.090 mg_Fe_/L. The effectiveness of removing iron compounds was approx. 30%, whereas in terms of lowering turbidity, it was approx. 33%. The content of organic substances measured as TOC, DOC, UV_254_, and UV_272_ also reduced. TOC concentration decreased from 10.110 to 9.468 mg_C_/dm^3^, DOC from 9.431 to 9.400 mg_C_/L, UV_254_ from 23.110 to 22.570 m^−1^, and UV_272_ from 19.060 m^−1^ to 18.310 m^−1^. Microfiltration is a physical method for the initial purification of water, rarely applied on a technical scale to pre-purify strongly contaminated surface waters; the effectiveness of this process depends, above all, on the size of the pores in the filtration material. The studies carried out on drum microfilters with the 10 µm filtration fiber pointed to the low usefulness of this process in removing organic substances. The effectiveness of removing TOC was 6.35%, DOC 0.33%, UV_254_ 2.34%, and UV_272_ 3.90%. Among the organic substances, those in an insoluble form were characterized by the highest levels of removal on microfilters. The size of particles in surface water fell in the range of 142 to 396 nm for water prior to microfilters and from 32.7 to 255 nm for water following microfilters (Figure 2).

Nearly 81% of particles found in surface water prior to microfiltering were those with a diameter ranging from 190 to 295 nm, whereas in water following the microfiltration process, 88% were those ranging from 32.7 to 78.8 nm.

#### 2.2.3. Coagulation 

**Figure 3:**
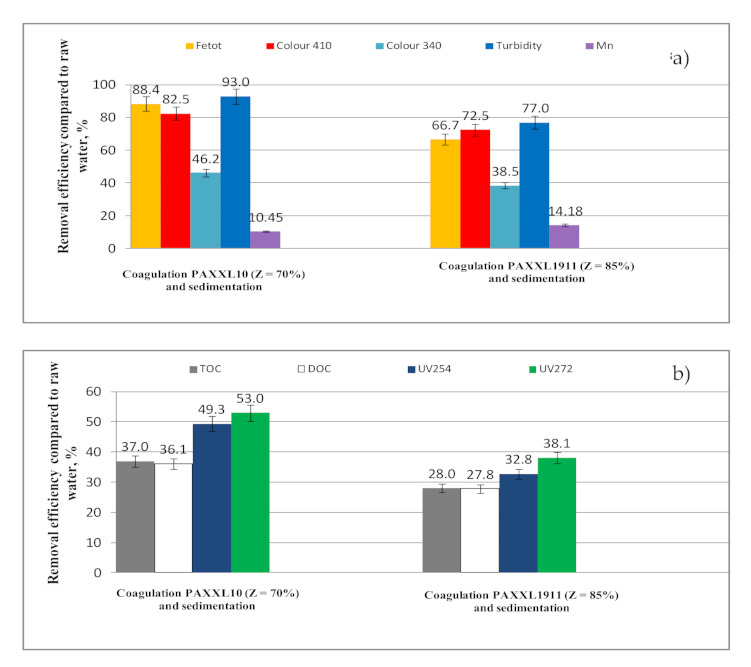
Efficiency of iron total, colour at wavelength 410 nm and 340 nm, turbidity, manganese (**a**) and TOC, DOC, UV_254_, UV_272_ (**b**) removal from water by coagulation.

The values of selected indicators of water quality after the coagulation process carried out on a technical scale using PAXXL10 (Z = 70%) polyaluminum chlorides and PAXXL1911 (Z = 85%, Fe = 0.7%) at a dose of 4 mg_Al_/L are presented in Table 2 and Table 3, with the efficiency of removing selected indicators of water quality during the coagulation process in regards to raw water presented in Figure 3a,b.

The analysis of the obtained test results showed that the PAXXL10 polyaluminum chloride of lower alkalinity (Z = 70%) characterized by smaller contents of polymerized forms (Al_b_ = 29%) ensured higher efficiency of purifying water than PAXXL1911 with an alkalinity of 85%, containing 40% of polymerized forms and, additionally, iron compounds in the amount of 0.7%. During the coagulation process carried out on a technical scale, iron compounds were removed to the greatest extent, as well as lowering the turbidity and color at a wavelength of 410 nm. The efficiencies of removing iron compounds, as well as lowering the turbidity and color at a wavelength of 410 nm for the more efficient PAXXL10 coagulant were: 88.4% (iron), 93% (turbidity), and 82.5% (color at a wavelength of 410 nm).

On the other hand, the efficiency of removing iron was significantly lower upon applying PAXXL1911 polyaluminium chloride, i.e., 66.7%, color at a wavelength of 410 nm 72.50% and turbidity 77% (Figure 3a). In the coagulation process, both using the PAXXL10 and PAXXL1911 coagulants, manganese compounds were removed to the lowest extent, with the efficiency of their removal found to be 10.45 and 14.18%, respectively. Following the PAXXL10 coagulation process, the concentration of iron as well as water turbidity were: 0.125 mg_Fe_/L and 1.05 NTU, and significantly higher upon coagulation using PAXXL1911, amounting to 0.360 mg_Fe_/L and 3.43NTU. After coagulation using PAXXL10 (Z = 70%), however, a nearly twice as high concentration of remaining aluminum was determined than following coagulation using PAXXL1911 (Z = 85%), with respective values of 0.362 and 0.172 mg_Al_/L (Table 2).

The analysis of the obtained research results also showed that the PAXXL10 coagulant (Z = 70%, Al_b_ = 29%) did more to lower the pH and alkalinity of the purified water than PAXXL1911 (Z = 85%, Al_b_ = 40%) as shown in Table 2. After the PAXXL10 coagulation process, the pH in purified water was 7.56 and the alkalinity was 3.40 mval/L, whereas after coagulation with PAXXL1911, pH was 7.80 and the alkalinity was 3.50 mval/L.

The calculated value of the SUVA_254_ indicator in raw mixed water was 2.471 m^2^/g_C_, which means that both hydrophilic, as well as hydrophobic compounds and small-particle organic compounds were present within, with a dominance of non-humic substances of small molecular weights. In accordance with the findings of the literature [34], the efficiency of removing organic substances, in this case, ought to be approx. 30%.

The analysis of the obtained results showed that, among all fractions of organic substances in the coagulation process, UV_272_ absorbance, thanks to which the share of THM precursors can be measured, was lowered to the greatest degree. Absorbance in UV_272_ was lowered by 53.00 and 38.15%, while absorbance in UV_254_ was lowered by 49.3 and 32.8% following respective coagulation using PAXXL10 (Z = 70%, Al_b_ = 29%) and PAXXL1911 (Z = 85%, Al_b_ = 40%, Fe = 0.7%) coagulants (Figure 3b). The measurement of absorbance at a wavelength of 254 nm allowed for identification of the DOC fraction containing aromatic rings, and, at the same time, characterized by a high potential of forming oxidation and disinfection by-products. The measurement of absorbance in UV_254_ is not, however, entirely selective, seeing as how absorption abilities at this wavelength also indicate nitrates, bromides, as well as organic substances with conjugated double bonds [31]. In connection with the above, in agreement with literature reports [32,35,36], a measurement at a wavelength of 272 nm, which is applied as an indicator of the potential of creating disinfection by-products (especially THM), is considered more specific.

The efficiency of removing TOC and DOC in the PAXXL10 coagulation process carried out on a technical scale was 37.0 and 36.1% and, after applying PAXXL1911 coagulant, 28.9 and 27.8%, respectively. Color measured at a wavelength of 340 nm indirectly characterizing the value of colorful organic substances was lowered in 46.2% (PAXXL10) and 38.5% (PAXXL1911)—Figure 3a. The value of TOC and DOC in water following the coagulation process was found to be 5.185 mg_C_/L and 5.000 mg_C_/L, and after coagulation using PAXXL1911, 5.926 mg_C_/L and 5.650 mg_C_/L, respectively.

Color measured at wavelengths of 410 and 340 nm after PAXXL10 coagulation, on the other hand, took values of 7 and 14 mg_Pt_/L, as compared to values of 11 and 16 mg_Pt_/L found following PAXXL1911 coagulation. Serving as confirmation of the effectiveness of the PAXXL10 coagulant (Z = 70%, Al_b_ = 29%), which does not contain iron in its composition, was also the measurement of the zeta potential in water samples after coagulation.

The value of the zeta potential in water samples following PAXXL10 coagulation was −5.30 mV, compared to −8.77 mV after PAXXL1911 coagulation.

**Figure 4:**
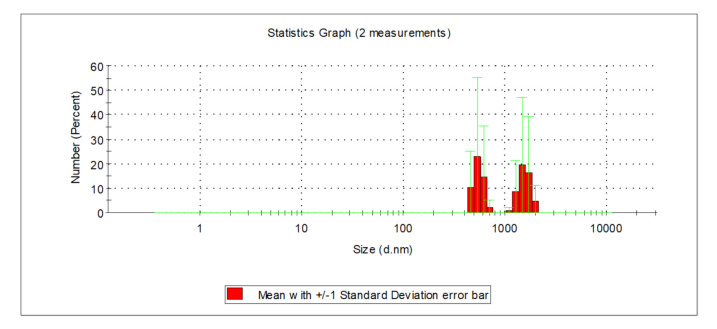
Distribution of particles in water following the PAXXL10 coagulation process.

**Figure 5:**
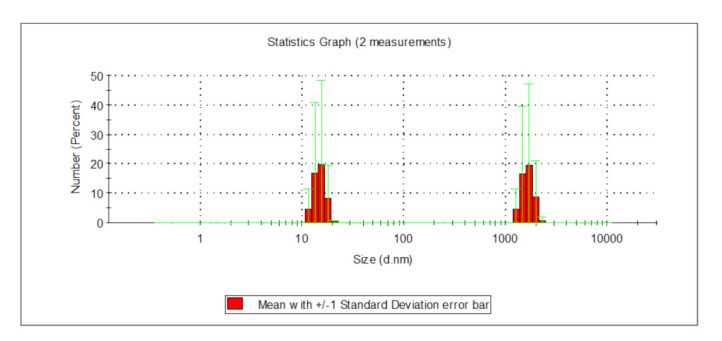
Distribution of particle size in water following the PAXXL1119 coagulation process.

The analysis of the relationships presented in Figure 4 and Figure 5 showed that particle size in water following the PAXXL10 coagulation process fell into the range of 459 and 712 nm, as well between 1110 and 1999 nm (Figure 4), whereas both very small particles 11.7 to 21 nm in size, as well as big particles measuring between 1280 and 2300 nm in diameter were found in water after the PAXXL1911 coagulation process (Figure 5).

Only in water after PAXXL10 coagulation was the value of the SUVA_254_ indicator lower than two, i.e., 1.960 m^2^/g_C_ (Table 3), thus confirming that there is no longer a necessity to continue the coagulation process due to fulfilling the production criteria for water that is deemed safe for the consumer, thanks to the organic precursors of oxidation and disinfection by-products having been sufficiently removed.

#### 2.2.4. Filtration Through a Catalytic-Oxidative Bed

**Figure 6:**
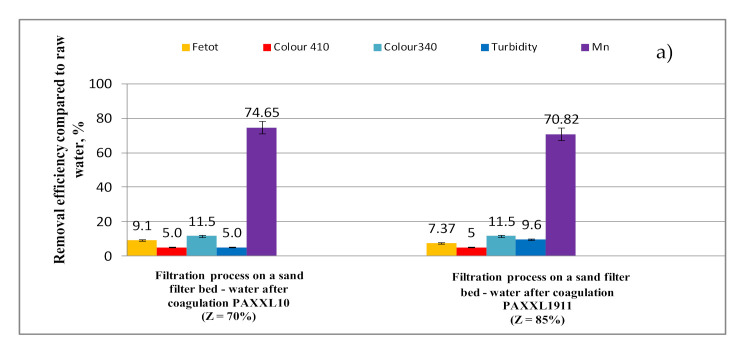

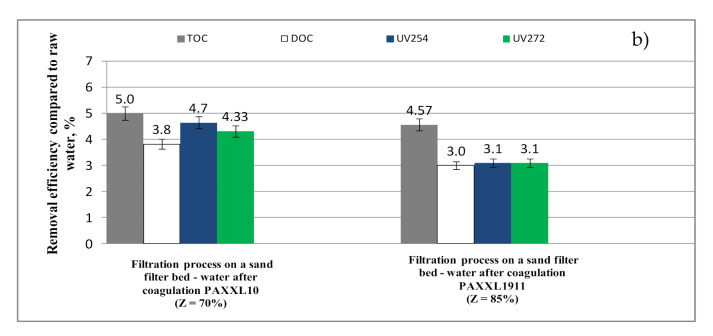
Efficiency of iron total, colour at wavelength 410 nm and 340 nm, turbidity, manganese (**a**) and TOC, DOC, UV_254_, UV_272_ (**b**) removal from water by sand filtration on catalytic-oxidative filter bed.

The analysis of research results presented in Figure 6a,b showed that, in the subsequent stage of water purification, during filtration on a catalytic-oxidative sand filter bed, manganese compounds were removed to the greatest extent, from 70.82 (following PAXXL1911 coagulation) to 74.55% (following PAXXL10 coagulation), which was to be anticipated due to the presence of manganese (IV) oxide in the catalytic-oxidative filter bed, which has adsorption abilities in regards to many metals, also including Mn(II), but, above all, plays the role of an oxidizing agent of Mn(II) ions to Mn(III), which are then oxidized with dissolved oxygen to Mn(IV) and precipitated from water as MnO_2_·H_2_O [37]. Irrelevant of the type of tested coagulant, the concentration of manganese in water after filtration through a catalytic-oxidative sand filter bed, was 0.02 mg_Mn_/L.

According to Michel et al. [38], methods based on oxidation on the surface of the filter bed in the presence of MnO_2_ are recommended because they reduce the addition of chemicals into the water, especially chlorine compounds, which prevents the formation of oxidation by-products.

The analysis of the obtained research results also showed that, during filtration on a catalytic-oxidative filter bed, the effectiveness of removing iron compounds, lowering the color and turbidity as well as removing organic substances increased comparatively, regardless of the tested coagulant, (Figure 6a,b). However, only after coagulation with PAXXL10 and filtration through a catalytic-oxidative filter bed were the concentrations of iron (≤0.2 mg_Fe_/L) and turbidity (≤1NTU) necessary for water to be suitable for human consumption [39], i.e., 0.027 mg_Fe_/L and 0.3 NTU, obtained. Following coagulation with PAXXL1911 and filtration through a sand filter bed, the concentration of iron in water was 0.280 mg_Fe_/L, with a water turbidity of 2 NTU.

After being subjected to the filtration process through a catalytic-oxidative filter bed, irrelevant of the type of applied coagulant, the required aluminum concentrations remaining in water intended for human consumption were obtained, i.e., 0.036 and 0.029 mg_Al_/L, respectively, for water following PAXXL10 and PAXXL1911 coagulation, and 5 and 11 mg_Pt_/L respectively, at a wavelength of 410 nm.

During filtration on a catalytic-oxidative filter bed, TOC was removed to the greatest extent of all the analyzed fractions of organic substances; the efficiency of its removal by means of a filtration process increased by 5.00 and 4.57% respectively for water following coagulation using PAXXL10 and PAXXL1911. The efficiency of removing DOC as a result of filtration through a catalytic-oxidative filter bed increased by 3.80% (following PAXXL10 coagulation) and 3.00% (following PAXXL1191 coagulation).

The concentrations of TOC and DOC in water after coagulation with PAXXL10 and filtration through a sand filter bed were 4.774 and 4.700 mg_C_/L, whereas after PAXXL1911 coagulation and filtration through a sand filter bed, their values were found to be 5.550 and 5.415 mg_C_/L.

Only in water after filtration through a catalytic-oxidative filter bed and PAXXL10 coagulation was the value of the SUVA_254_ indicator lower than 2, i.e., 1.840 m^2^/g_C_ (Table 3), thus meeting the criteria for the production water that is safe for the consumer in terms of the presence of precursors of oxidation and disinfection by-products in the treated water.

After the filtration process on the catalytic-oxidative filter bed, the presence of particles with sizes ranging from 142 to 825 nm was confirmed, irrelevant of the type of applied coagulant. Therefore, the filtration process on a catalytic-oxidative filter bed removed mainly very large particles—over 1000 nm in diameter—and very small ones—up to 20 nm. Nearly 80% of all particles present in the water following filtration on a sand filter were in the range from 342 to 531 nm (Figure 4 and Figure 5 andFigure 7).

#### 2.2.5. Filtration Through a Dolomite Bed

The second level of filtration on filters with a dolomite filter bed caused an increase in pH to a value of 7.75, as well as alkalinity to a value of 3.45 mval/L in the case of water purified with the PAXXL10 coagulant; in the case of water purified with the PAXXL1911 coagulant, the pH has increased to 7.95 after filtration on a dolomite filter bed, with alkalinity reaching 3.50 mval/L (Table 2).

**Figure 8:**
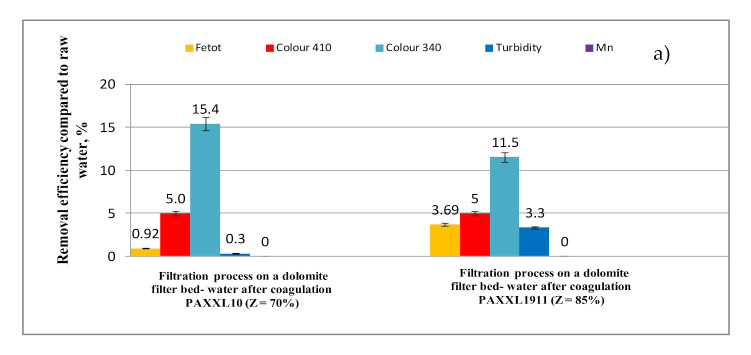

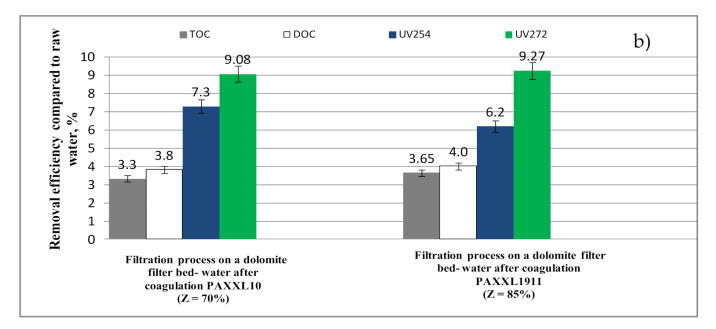
Efficiency of iron total, colour at wavelength 410 nm and 340 nm, turbidity, manganese (**a**) and TOC, DOC, UV_254_, UV_272_ (**b**) removal from water by filtration with a dolomite bed.

In the filtration process on a dolomite filter bed, color measured at a wavelength of 340 nm was lowered to the greatest degree, as well as the content of dissolved organic substances containing aromatic rings, which were measured using UV_254_ and UV_272_ absorbance (Figure 8). The efficiency of lowering color at a wavelength of 340 nm as a result of filtration through a dolomite filter bed increased by 15.4 and 11.5% for water purified with the respective PAXXL10 and PAXXL1911 coagulants. The efficiency of lowering UV_254_ and UV_272_ absorbance as a result of filtration through a dolomite filter bed increased by 7.30 and 9.08% respectively in water purified with the PAXXL10 coagulant, and 6.20 and 9.27% for water purified with PAXXL1911 coagulant. 

In accordance with reports found in the literature [40], the processes of removing organic substances from water as a result of filtration through a dolomite filter bed, especially removing organic substances containing aromatic rings, such as humic substances, is connected with the ability of these substances to create complexes with calcium and magnesium ions.

The complexes formed by calcium and magnesium ions with organic substances are formed as a result of the ion exchange of the H^+^ cation to a metal ion or as a result of the formation of coordinate bonds by –COOH and –OH functional groups containing oxygen, which play the role of a ligand as well as partially by ionic bonding. Complexes with a majority of coordinate bonds are more permanent than those with a majority of ionic bonds.

The complexing reaction takes place gradually, i.e., ligands attach to the central atom creating a series of intermediate complexes, prior to the formation of a coordinatively saturated complex. The strongest bond is between the metal and the first coordinated ligand. An increase in strength is caused by a rising dissociation of –COOH and –OH functional groups at a pH of approximately 8, as well as the high competitiveness of H^+^ ions in relation to metals in an acidic environment [41].

In water samples after the second stage of filtration on a dolomite filter bed, a further decrease in the zeta potential to respective values of −4.50 and −7.00 mV was confirmed in water samples that had undergone PAXXL10 and PAXXL1911 coagulation. Decreasing the zeta potential was most likely an effect of the reaction between disassociated functional groups of organic substances and calcium and magnesium ions from the dolomite filter [42].

The analysis of the obtained results showed that, after the second stage of filtration on a dolomite filter bed, water quality parameters of water intended for human consumption were not achieved only in the case of water purified with the PAXXL1911 coagulant with an alkalinity of 85% and containing iron compounds in its composition; this was due to exceeded levels of turbidity, iron, and total organic carbon, which were characterized by respective values of 1.50 NTU, 0.24 mg_Fe_/L, and 5.250 mg_C_/L.

Moreover, the value of the SUVA_25_ indicator in such water was higher than 2 and reached 2.190 m^2^/g_C_ (Table 3), thus not meeting the production criteria for water deemed safe for the consumer due to the presence of precursors of oxidation and disinfection by-products in the treated water.

**Figure 9:**
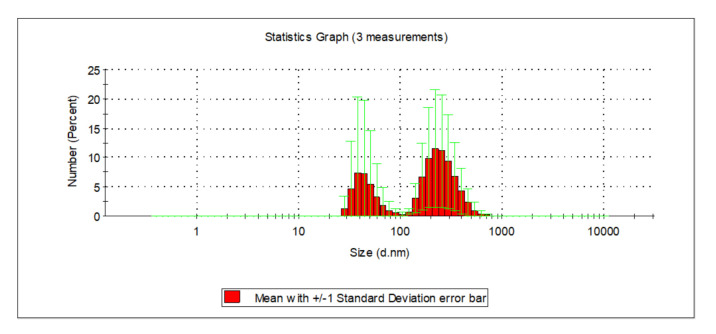
Distribution of particle sizes in water following the filtration process on a dolomite bed.

After the filtration process had been carried out on a dolomite bed, the presence of particles whose size fell in the range from 122 to 825 nm was indicated regardless of the type of applied coagulant. Nearly 80% of all particles present in the water following the filtration process on a dolomite bed had diameters in the range of 190 to 459 nm (Figure 9).

#### 2.2.6. Disinfection

**Figure 10:**
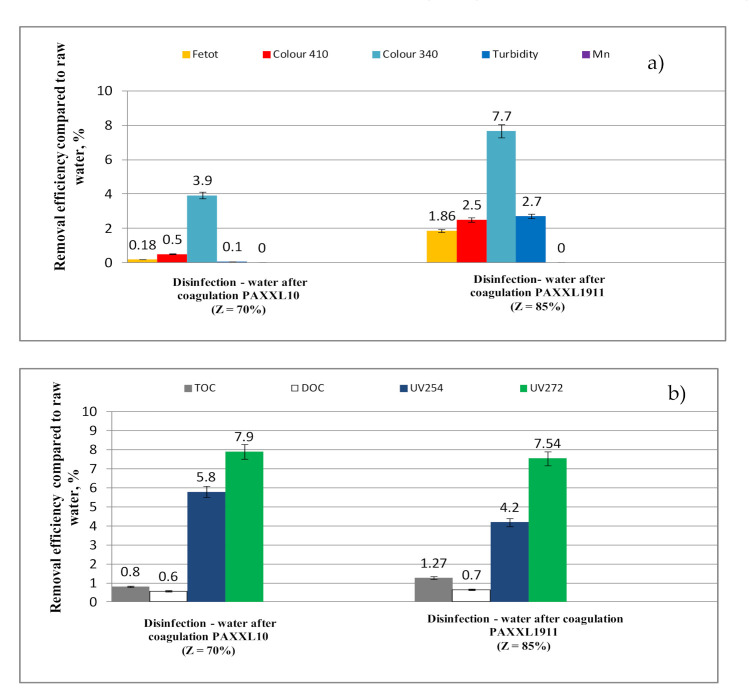
Efficiency of iron total, colour at wavelength 410 nm and 340 nm, turbidity, manganese (**a**) and TOC, DOC, UV_254_, UV_272_ (**b**) removal from water by disinfection.

The process of disinfection using chlorine dioxide caused the partial oxidation of organic substances containing aromatic rings, as confirmed by the greatest decrease in absorption measured at wavelengths of 254 and 272 nm, which was 5.80% (UV_254_) and 7.90% (UV_272_), respectively, for water following coagulation with PAXXL10, and 4.20% (UV_254_) and 7.54 % (UV_272_) for water following coagulation with PAXXL1911 (Figure 10b).

In the disinfection process, the color of water measured mainly at a wavelength of 340 nm was also found to have decreased, confirming the presence of colored organic substances with chromophoric groups. In water that had been subjected to coagulation with PAXXL10, the efficiency of decreasing color at a wavelength of 340 nm in the disinfection process was 3.90%, while after coagulation with PAXXL1911, this percentage was 7.70%.

In the disinfection process, a greater increase in the effectiveness of removing iron, color, and turbidity was also observed in water post PAXXL1911 coagulation than in water after coagulation with PAXXL10. Nevertheless, parameters of the water quality suitable for human consumption were not achieved following the disinfection process after coagulation with PAXXL1911 due to the turbidity, iron concentration, and TOC, which were minimally exceeded and characterized by values of 1.10 NTU, 0.22 mg_Fe_/L, and 5.145 mg_C_/L.

The calculated value of the SUV_254_ indicator in water following the disinfection process and coagulation with PAXXL1911 was 2.060 m^2^/g_C_ as compared to 1.580 m^2^/g_C_ after coagulation with PAXXL10.

**Figure 11:**
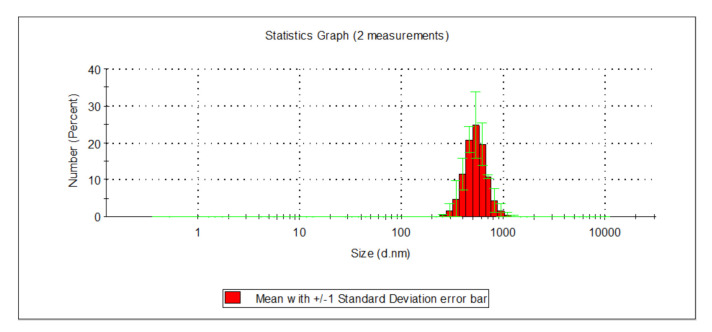
Distribution of particle sizes in water after the disinfection process.

Following the process of disinfection in purified water, regardless of the type of coagulant applied, the presence of particles with a diameter ranging 255 to 955 nm was confirmed (Figure 11). In the disinfection process, particles below 255 nm in diameter were oxidized, which was confirmed in water that had undergone the filtration process on a dolomite bed.

In accordance with the findings of the literature, the reactions of chlorine dioxide with organic substances take a long time and occur mainly in the water distribution system; the by-products of oxidation may, in turn, increase the concentration of biodegradable organic carbon, facilitating the successive growth of microorganisms.

Chlorine dioxide, thanks to its radical structure is, above all, an acceptor of electrons and thus an oxidizer; in contrast to chlorine, it does not stimulate bonding or substitution reactions, and thus the chlorination reaction [42,43]. The by-products of the oxidation of organic substances, in this case, are mainly aldehydes and low molecular organic acids, but also compounds of undetermined structures. The reaction with chlorine dioxide leads mainly to the oxidation of particles whose molecular weights range from 3500–500 Da, to oxidation by-products with molecular weights below 300 Da [44].

### 2.3. Jar Test

Before carrying out studies on a technical scale using two polyaluminum chlorides, i.e., PAXXL10 (Z = 70%) and PAXXL1911 (Z = 85%, Fe = 0.7%), laboratory studies of the coagulation process using the jar test were also carried out, applying the same coagulants in doses from 1 to 5 mg_Al_/L. The subject of the studies was also the same mixture of the surface water from the Obrzyca River after microfiltration, as well as groundwater subjected to the aeration process from the Zawada water treatment plant.

#### Removing Iron and Organic Substances from Water

The analysis of the obtained results showed that, similarly to the studies carried out on a technical scale, a higher effectiveness of water purification was obtained when applying the coagulant PAXXL10 with an alkalinity of 70% rather than PAXXL1911, characterized by an alkalinity of 85% and containing iron compounds in its composition.

The pH of water after coagulation with the PAXXL1911 coagulant (Z = 85%) ranged from 7.85 to 7.96, and in water following PAXXL10 coagulation (Z = 70%), from 7.50 to 7.80 for doses from 1 to 5 mg_Al_/L. In water samples following PAXXL1911 coagulation, the concentration of iron, as well as turbidity for doses from 1 to 5 mg_Al_/L, ranged from 0.306 to 0.221 mg_Fe_/L and from 3.20 to 2.34 NTU, and were significantly greater than after PAXXL10 coagulation. On the other hand, the concentration of iron and turbidity of the water after coagulation with the PAXXL10 coagulant took on values ranging from 0.140 to 0.082 mg_Fe_/L and from 2.11 to 0.91NTU.

The share of iron (II) in the total iron content after coagulation with PAXXL1911 was from 29 to 19% and decreased along with an increasing dose of the coagulant. The share of iron (II) in the total iron following coagulation with PAXXL10, on the other hand, amounted to 22 to 24%, and increased with the rising dose of the coagulant, along with a decrease in pH during the coagulation process, which was found to be in the range from 7.50 to 7.80, as compared to PAXXL10 coagulation (pH from 7.85 to 7.96).

In water samples following PAXXL1911 coagulation, a higher color measured at wavelengths of 340 and 410 nm, as well as higher concentrations of organic substances measured as TOC, DOC, as well as UV_254_ and UV_272_ were noted. The color measured at a wavelength of 410 nm after coagulation with PAXXL1911 changed in the range of 18 to 14 mg_Pt_/L, whereas at a wavelength of 340 nm—in the range from 24 to 16 mg_Pt_/L. The concentration of TOC changed from 7.221 to 5.799 mg_C_/L, DOC from 6.919 to 5.595 mg_C_/L, UV_254_ from 17.73 to 13.20 m^−1^, and UV_272_ from 14 to 10.32 m^−1^.

The calculated value of the SUVA_254_ indicator in water after PAXXL1911 coagulation for doses from 1 to 5 mgAl/L ranged from 2.56 to 2.36 m^2^/g_C_, and thus, as was the case during technological studies, the criteria for the production of water safe for consumers were not met in the case of this coagulant due to the presence of precursors of oxidation and disinfection by-products in the treated water. The value of the SUVA_254_ indicator in water after PAXXL10 coagulation for doses from 1 to 5 mg_Al_/L changed within the range from 2.33 to 1.80 m^2^/g_C_. The analysis of the obtained study results also showed that the concentration of aluminum formed in water following coagulation with PAXXL10 was significantly higher than in water that had been subjected to PAXXL1911 coagulation and exhibited respective values from 0.260 to 0.370 mg_Al_/L (PAXXL10), as well as from 0.030 to 0.180 mg_Al_/L (PAXXL1911). The analysis of relationships presented in Table 4 showed that, in water following coagulation with PAXXL1911, linear correlations were confirmed between iron (III), turbidity, color, and organic substances, measured as TOC, DOC, and UV_254_. This confirms that colored iron-organic connections were created in the water during coagulation. 

According to numerous researchers [5,6,7,8,9,45], one of the reasons for the stabilization of iron by organic substances is the formation of chelate complexes and the formation of so-called protective colloids of a hydrophilic nature. After the coagulation process with PAXXL1911 coagulant with an alkalinity of 85% containing iron additives in its composition, the highest values of the Pearson coefficient were confirmed between iron (III) and dissolved organic substances containing aromatic rings, as well as between turbidity and iron (III), and also between the color measured at a wavelength of 340 nm and DOC and UV_254_. According to Kwakye-Awuah et al. [37] the iron ion pollution in groundwater may interact with the dissolved constituents, such as dissolved organic carbon (DOC), present as a humic-like material. These interactions lead to complex formations and to potential solid precipitation.

The reason behind the formation of chromatic ferroorganic connections over the course of coagulation with the highly-alkaline PAXXL1911 coagulant may have been the high pH, falling into the range of 7.96 to 7.85, in which the functional groups of –COOH and –OH organic substances are more reactive in relation to iron ions.

The second reason may have been the introduction of additional iron ions along with the coagulant, which may have reacted with the organic substance present in the treated water. The colored iron-organic connections formed during PAXXL1911 coagulation most likely exhibited the character of protective colloids, hence leading to the significantly higher turbidity that followed PAXXL1911 coagulation as compared to the turbidity after PAXXL10 coagulation. During the research conducted by Albertkiene [46] with the use of groundwater containing iron-organic complexes, it was shown that water pH has the greatest impact on the removal of iron-organic complexes from drinking water during coagulation. Iron-organic complexes are best eliminated from drinking water when its pH is from 6.8 to 6.5, a rise in pH reduces the effectiveness of their removal.

**Figure 12:**
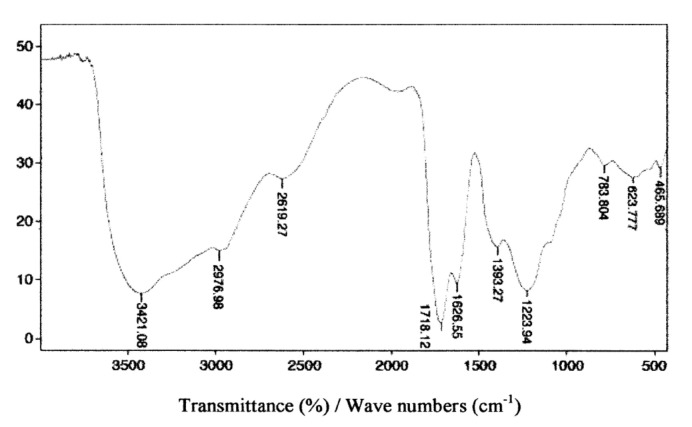
Spectrum of fulvic acids extracted from colloids present in water following the coagulation process using the highly alkaline PAXXL1911 coagulant.

According to many authors [5,6,7,8,9,10,11], one of the causes of iron stabilization by organic substances in underground waters may be the formation of so-called protective colloids of a hydrophilic nature, created as a result of the adsorption of organic substances on the surface of iron (III) hydroxide. The organic stabilization of iron colloids results from the formation of an external encasement containing ionized carboxyl groups. The notion that iron may create mixed complexes with organic and non-organic ligands in natural waters is also deemed feasible. It was moreover confirmed that the constant durability of the ferroorganic complex increases along with an increase in pH, which is most likely caused by a growing dissociation of functional –COOH and –OH groups, as well as the lower competitiveness of H^+^ ions in relation to metals, which took place in an acidic environment. The interaction of iron with colloids may result in iron–organic connections which are colloidal and soluble in water to various degrees. The analysis of the IR spectrum confirmed that fulvic acids, which, in comparison to humic acids have more carboxyl and aliphatic group vibrations [47,48,49,50], were present in colloids isolated from water following the process of coagulation using highly alkaline polyaluminum chloride PAXXL1911 (Z = 85%, Fe = 0.7%) (Figure 12).

An interpretation of the bands includes the O–H stretch vibration caused by alcoholic, phenolic, and acid groups at ~3400 cm^−1^. The peak, at ~2977 cm^−1^, is caused by C–H stretching vibrations of the H_3_C– and CH_2_– groups. Absorption at 2619 cm^−1^ is associated with the OH stretch of the carboxylic groups. The presence of carbonyl groups in carboxylic acids, esters, ketones, and aldehydes structures gives rise to the C=O stretching vibrations at ~1720 cm^−1^. Absorption at ~1620 cm^−1^, on the other hand, stems from stretch vibrations of C=C bonds in aromatic structures, whereas that at ~1400 cm^−1^ corresponds to O–H bonding vibrations of alcohols and carboxylic acids. The range below 1280 cm^−1^ is dominated by C–O stretching vibrations and O–H deformations in carboxylic acids.

## 3. Materials and Methods

The research was conducted on a laboratory and technical scale. During the research of studies carried out on a laboratory scale, the efficiency of the coagulation process was carried out with the use of the jar test. As a coagulant, the commercial average and highly alkaline polyaluminium chlorides were tested because the amount of monomers and polymers in polyaluminium chlorides solutions varies and depends mainly on the value of the alkalinity coefficient of coagulants (r = [OH^−^]/[Al^3+^]). It was also assumed that the increase in pH during the coagulation process with highly-basic polyaluminium chlorides and the presence of iron in the composition of coagulant, as well as in the raw water, may result in the formation of iron–organic complexes. The same coagulants were also tested on a technical scale. The aim of the studies conducted on the technical scale was to determine whether the type of coagulant that may contribute to the formation of iron–organic complexes during the coagulation process has an impact on the final effectiveness of water treatment.

The research on a technical scale was carried out at a water treatment plant in "Zawada" near Zielona Góra in Poland. During the research of studies carried out on a technical scale the efficiency of the following processes was assessed: microfiltration, aeration, coagulation and sedimentation, filtration through a catalytic-oxidative sand bed covered with iron and manganese oxides, filtration through a dolomite filter bed, and disinfection with chlorine dioxide.

### 3.1. Water Samples

The subject of the study was a mixture of surface water from the Obrzyca River and the groundwater after the aeration process from Quaternary formations. Groundwater, following the aeration process, was mixed with surface water subsequent to the process of microfiltration on drum microfilters at a volume ratio of 1:2.

At the "Zawada" water treatment plant, groundwater from Quaternary formations and surface water from the Obrzyca River were collected. Due to the continuing hydrogeological drought, the amount of groundwater taken in was limited, and therefore, a mixture of groundwater and surface water was treated. The intake of water from the Obrzyca River was 15,000 to 22,000 m^3^/day and the capacity of groundwater intake did not exceed 5000 m^3^/day.

### 3.2. Jar Test

The tests of coagulation were carried out by a 1 L six-place paddle stirrer (Flocculator Kemira 2000, Helsingborg, Sweden). Coagulation was carried out in water samples of 1 L through 1 min fast mixing at a speed of 250 rpm and 25 min flocculation with an intensity of mixing of 30 rpm. As a coagulant, the commercial pre-hydrolyzed polyaluminium chloride PAXXL10 with an alkalinity of 70% and alkalinity coefficient of r = 2.10, as well as PAXXL1911 polyaluminium chloride with an alkalinity of 85% and alkalinity coefficient of r = 2.55, which, in its composition, also contained iron in the amount of approx. 0.7%. were tested (Table 5). Polyaluminium chlorides are produced by the KEMIPOL company in Police (Poland). The doses of coagulants were expressed in mg_Al_/L and varied from 1 to 5 mg _Al_/L. After coagulation, the samples were subject to the sedimentation process for 1 h.

The characteristics of the tested coagulants are presented in Table 5, accounting for the alkalinity as well as results of the specification analysis of aluminum. The share of individual forms of aluminum was determined using the method of classical ferronometry [24,51]. The analysis of the obtained research results showed that the PAXXL1911 coagulant contained a higher amount of polymer forms of aluminum (Al_b_), amounting to as much as 40%, than the PAXXL10 coagulant, in which the content of polymer forms of aluminum was 29%.

### 3.3. Characteristics of Water Treatment Technology

**Figure 13:**
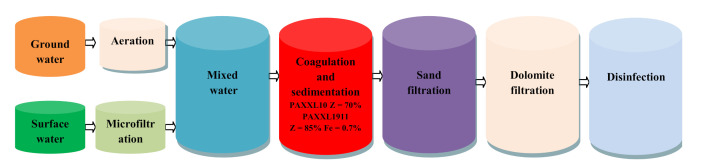
Water treatment scheme applied in the “Zawada” Water Treatment Plant in Zielona Góra.

During research conducted on a technical scale, water samples for carrying out physical and chemical analyses were collected after subsequent stages of purifying the water—a mixture of groundwater and surface water. Sample collection was carried out weekly in the summer for a period of two months. The efficiency of microfiltration was determined in relation to surface water (which, upon mixing with groundwater following aeration, comprises the resource for this plant). The water treatment process is carried out in two of the same process lines. The maximum output of each production process is 750 m^3^/h. The scheme of the water treatment process train applied in the “Zawada” Zielona Góra water treatment plant is presented in Figure 13.

The aeration process of groundwater takes place in four cascades, with forced airflow with a maximum output of 220 m^3^/h each. Water, following aeration, flows into two reservoirs with a capacity of approx. 250 m^3^ each.

Surface water, after being subjected to microfiltration using microscreen drum filters with 10 µm pores and a coagulation process, was also channeled to these chambers, as well as the returned post-coagulation sludge.

High-speed mixers are found in fast mixing chambers, which mix both the waters and the coagulant. The post-coagulation sludge is partially returned to the coagulation process with the aim of improving the conditions in which floccules form and increasing the effectiveness of the process. Water mixed with the coagulant flows into two serially connected slow-mix chambers. Stirrers with diagonally constructed paddles are found in the slow-mix chambers, giving the water in the chambers horizontal and vertical rotational movement. The speed of mixing is regulated and amounts to a maximum of 20 rotations/min. The sediments which are formed in the contact chambers and mixing chambers are carried away gravitationally to process wastewater collectors. The sludge is discharged periodically according to need. Water with formed post-coagulation floccules flows into the chambers of the sedimentation tanks. In the first part of the sedimentation tanks, the clarification process takes place as it does in the horizontal sedimentation chambers, whereas in the second part, lamella separators are integrated with the system of receiving troughs. The sediment which collects at the bottom of the chamber is gathered into sludge hoppers with the use of mechanical sweepers.

During technological studies carried out on a technical scale, the following were tested as coagulants: PAXXL10 with an alkalinity of 70% as well as PAXXL1911 polyaluminium chloride with an alkalinity of 85% which, in its composition, also contained iron (Table 5). The doses of tested coagulants were 4 mg_Al_/L.

The subsequent stage in the water purification process is filtration using open rapid filters through a sand filter bed covered by manganese (IV) and iron (III) oxides i.e., the so-called catalytic-oxidative filtration bed. Following the filtration process on the catalytic-oxidative filter bed, water is additionally purified during the second stage of filtration on dolomite filters, where the filter bed is activated dolomite (CaCO_3_·MgO). Disinfectant, which is a chlorine dioxide solution, is added to the purified water flowing into the tank. In Table 6, the sample collection points have been presented. The effectiveness of the coagulation and sedimentation process has been determined together as the effectiveness of coagulation.

### 3.4. Analytical Methods

The physical-chemical composition of both the raw water as well as treated water was determined according to the International Standard methods. The organic substances in all samples were determined by measuring total (TOC) and dissolved organic carbon (DOC) concentration, color (absorbance of 410 and 320 nm wavelength), and absorbance at 254 and 272 nm. TOC (DOC) concentration is the most reliable method for determining the total amount of NOM, UV absorption at 254 nm monitors the amount of NOM fractions containing aromatic structures in their molecules [31,32]. The TOC and DOC were measured using the thermal method and a Shimadzu TOC analyzer. DOC was analyzed by the TOC Analyzer after filtration through 0.45 µm pore diameter membranes. UV absorbance at 254 nm (UV_254_) and at 272 nm (UV_272_) was measured by a UV-VIS spectrophotometer Agilent Cary 60 using a quartz cell with 1 cm path length after filtration through a 0.45 µm membrane. DOC and UV_254_ are used in the calculation of the specific UV absorbance (SUVA_254_).

SUVA_254_ = UV_254nm_/DOC (m^2^/g_C_) (1)

where SUVA is specific UV absorbance at 254 nm (m^−1^) and DOC is dissolved organic carbon (g_C_/m^3^) [31].

True color was indicated in accordance with ISO 7887-Method C [33], using a spectrophotometer Agilent Cary 60. Quartz cuvette with a path length of light 50 mm was used. It was determined after filtration of the water sample through a membrane filter of pore size 0.45 μm. The total iron and iron (II) concentrations were determined with the Dr 3900 (HACH Lange) spectrophotometer. Iron (II) was measured using the 1,10 phenanthroline method. Total iron was measured using the same method. As a reducing agent of ferric ions to the ferrous ions, hydroxylamine hydrochloride was used. Aluminum concentration was determined with the atomic emission spectroscopy (ISP-OES, 5300DV, Perkin Elmer Company, Waltham, Massachusetts US). The pH of the raw water and the purified water was determined with a WTW Multi Line P4 with a combination pH electrode with temperature corrections. Turbidity was measured using the Turbidimeter 2100N, Hach Company, Loveland, Kolorado, USA. The alkalinity was determined with a titrimetric method against methyl orange using 0.1 M aqueous solutions of HCl. The Al species distribution in the PACls (PAXXL10, PAXXL19110) samples was analyzed by the Ferron complexation timed spectrophotometry [51]. Al^3+^ reacts with Ferron reagent to form an Al-Ferron complex at pH = 5, λ = 370 nm. An Agilent Cary 60 (Santa Clara, California, USA) spectrophotometer was used to measure the Al-Ferron kinetics. Based on the kinetic difference of reactions between Ferron reagent (8-hydroxy-7-iodo-5-quinoline sulfonic acid) with different hydrolyzed species, hydrolyzed Al species can be divided into three types: monomeric Al species (Al_a_) (instantaneous reaction: 0 to 1 min), medium polymerized Al species (Al_b_) (reaction within 120 min), and species of colloidal (Al_c_) (no reaction in 120 min). The results are shown in Table 1. Measurement of the electrokinetic potential ζ was made in water samples using the Zetasizer Nano Analyzer (Malvern Panalytical Company, Cambridge, UK), which calculates the Zeta potential by determining the electrophoretic mobility of the particles using the laser technique of speed measurement based on the Doppler effect. In the water, the particle size was also measured using the Zetasizer Nano Analyzer. The Zetasizer Nano Analyzer measures particle size using the dynamic light scattering (DLS, dynamic light scattering) process, also known as photon correlation spectroscopy (PCS, photon correlation spectroscopy), which measures Brown’s motion and calculates particle size on this basis. The intensity of the fluctuation of the scattered laser light that the particles are illuminated by was analyzed. Water samples following the coagulation process using PAXXL10 and PAXXL1911 were filtered through a filter 0.45 µm in diameter. Humic substances were extracted from the colloids isolated from water following the PAXXL10 and PAXXL1911 coagulation process according to the methodology developed by Aiken et al. [34,35,36], using different solubilities in acids and alkalis for individual fractions of humic substances. Humic substances extracted from raw water were analyzed using infrared (IR) spectroscopy using an FTIR Thermo Scientific Nicolet iS50 spectrometer (Waltham, MA, USA) operating in the NIR-, MID-, and FAR-IR ranges using the KBr compensation tablet technique.

## 4. Conclusions

The analysis of the test results leads to the following conclusions:

1. Iron compounds, as well as organic substances, but also color and turbidity, were removed at the highest levels in the coagulation process, which also had a significant effect on the final effectiveness of water purification. The efficiency of iron removal during the coagulation process was 66.7 and 88.4%, and of organic substances: 28.0 and 37.0% (TOC), 27.8 and 36.1% (DOC).
2. The effectiveness of purifying water in the coagulation process was determined by the type of tested polyaluminium chloride. Polyaluminium chloride PAXXL1911, which is characterized by a higher alkalinity and additionally contains iron ions in its composition, caused a significantly lower effectiveness of removing iron compounds (66.7%) and organic substances (28.0%-TOC, 27.8%-DOC), and also decreasing the color (72.5%-color at wavelength 410 nm; 38.5%-color at wavelength 340 nm), and turbidity (77.0%) as well as the zeta potential (23.0%), despite the fact that it contained higher amounts of polymer forms of aluminum (Al_b_ = 40%) than PAXXL10 (Al_b_ = 29%). The reason behind the lower effectiveness of water purification in the coagulation process upon applying PAXXL1911 was the formation of iron-organic connections, most likely in the form of protective colloids.
3. The reason behind the formation of colored iron–organic connections during coagulation with the highly alkaline PAXXL1911 coagulant may have been the high pH of approx. 8, at which the functional groups of organic substances are more reactive in relation to the metal ions, especially to iron, and the introduction of additional iron ions along with the coagulant which may have reacted with the organic substances present in the purified water, contributing to the formation of additional iron–organic connections.
4. Over the course of filtration on a catalytic-oxidative filter bed, the effectiveness of removing iron compounds (7.37 and 9.10%), as well as decreasing color (5.0%-color at wavelength 410 nm; 11.5%-color at wavelength 340 nm) turbidity (5.0 and 9.6%) and organic substances (4.57 and 5.00%-TOC; 3.00 and 3.80%-DOC) was also increased, though manganese compounds were removed to the largest extent (70.82 and 74.65%).
5. The second level of filtration on a dolomite bed mainly increased the effectiveness of removing organic substances containing aromatic rings (6.20 and 7.30%-UV_254_; 9.08 and 9.27%-UV_272_), as well as lowering the color measured at a wavelength of 340 nm (11.5 and 15.4%), at which they absorb mainly organic substances containing chromophoric groups. The processes of removing organic substances from water as a result of filtration through a dolomite bed, and especially removing organic substances containing aromatic rings, is connected with the ability of these substances to create complexes with calcium and magnesium ions.
6. The disinfection process using chlorine dioxide caused the partial oxidation of organic substances containing aromatic rings (4.20 and 5.80%-UV_254_; 7.54 and 7.90%-UV_272_). The analyses of the obtained results also showed that only after the disinfection process in water purified with highly-alkaline polyaluminium chloride (Z = 85%) containing iron compounds in its composition, water quality parameters of water intended for human consumption were not met due to iron (0.22 mg_Fe_/L), turbidity (1.10 NTU) and total organic carbon (5.145 mg_C_/L), while the calculated value of the SUVA_254_ indicator was over 2, thus not fulfilling the production criterion for safe water, as a result of the presence of precursors of oxidation and disinfection by-products. Therefore, the use of highly alkaline polyaluminum chlorides, which additionally contain iron ions in their composition, should not be recommended for the treatment of water containing excessively high concentrations of organic substances, including humic substances, due to the possibility of creating colored iron-organic connections difficult to remove in the entire technological system.

## Figures and Tables

**Figure 7:**
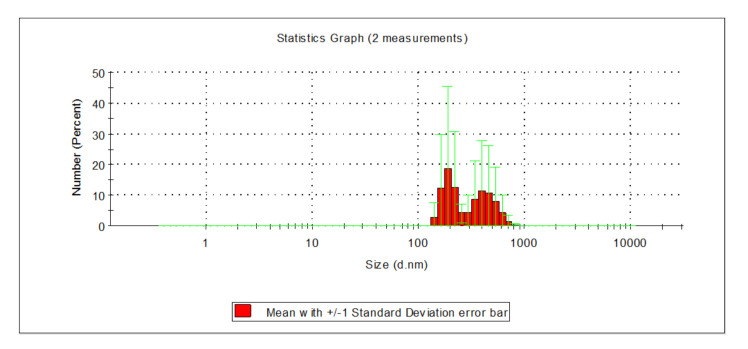
Distribution of particle sizes in water after the filtration process on a catalytic-oxidative sand filter bed.

**Table 1:** Physical and chemical characteristics of water collected at the Zawada water treatment plant.

Indicator	Unit	Value				
		Groundwater	Groundwater Following Aeration	Surface Water	Surface Water Following Microfilters	Mixed Water
pH	-	7.31 ± 0.1	7.70 ± 0.1	8.26 ± 0.1	8.30 ± 0.1	8.10 ± 0.1
Alkalinity	mmol/L	3.40 ± 0.05	3.40 ± 0.05	3.60 ± 0.05	3.60 ± 0.05	3.50 ± 0.05
Turbidity	NTU	15.00 ± 0.1	18.00 ± 0.1	2.10 ± 0.1	1.40 ± 0.1	15 ± 0.1
Colour 340 nm	mg_Pt_/L	20 ± 1	22 ± 1	36 ± 1	35 ± 1	26 ± 1
Colour 410 nm	mg_Pt_/L	23 ± 1	34 ± 1	48 ± 1	48 ± 1	40 ± 1
TOC	mg_C_/L	6.000 ± 0.05	6.000 ± 0.05	10.110 ± 0.05	9.468 ± 0.05	8.231 ± 0.05
DOC	mg_C_/L	5.800 ± 0.05	5.800 ± 0.05	9.431 ± 0.05	9.400 ± 0.05	7.827 ± 0.05
UV_254_	m^−1^	14.49 ± 0.200	14.49 ± 0.200	23.110 ± 0.200	22.570 ± 0.200	19.338 ± 0.200
UV_272_	m^−1^	12.94 ± 0.200	12.94 ± 0.200	19.060 ± 0.200	18.310 ± 0.200	16.165 ± 0.200
Iron _total_	mg_Fe_/L	2.353 ± 0.100	2.353 ± 0.100	0.128 ± 0.100	0.090 ± 0.100	1.080 ± 0.100
Iron (II)	mg_Fe_/L	1.900 ± 0.100	0.091 ± 0.100	0.078 ± 0.100	0.078 ± 0.100	0.085 ± 0.100
Iron (III)	mg_Fe_/L	0.453 ± 0.100	2.262 ± 0.100	0.05 ± 0.010	0.012 ± 0.100	0.995 ± 0.010
Iron (II)/Iron _total_	%	81	4	61	87	8
Elektrokinetic Potential	mV	−14.3	−12.2	−10.47	−10.47	−11.4
Manganese	mg_Mn_/L	0.240 ± 0.020	0.240 ± 0.020	0.086 ± 0.020	0.063 ± 0.020	0.134 ± 0.020
D = TOC/Iron _total_	mg_C_/mg_Fe_	2.549	2.549	78.984	105.2	7.621
D’ = DOC/Iron _total_	mg_C_/mg_Fe_	2.465	2.465	73.679	104.4	7.247
SUVA_254_	m^2^/g_C_	3.144	3.144	2.450	2.401	2.471

**Table 2:** Physical and chemical properties of water after individual stages of its treatment.

Sampling Point	pH	Alkalinity mval/L	Turbidity NTU	Iron_total_ mg/L	Iron (II) mg/L	Mn mg/L	Al mg/L
Mixed water	8.10	3.50	15.00	1.080	0.085	0.134	0
After PAXXL10 coagulation and sedimentation	7.56	3.40	1.05	0.125	0.008	0.120	0.362
After PAXXL1911 coagulation and sedimentation	7.80	3.50	3.43	0.360	0.017	0.115	0.172
After the filtration process on a catalytic-oxidative sand filter bed (PAXXL10 coagulation)	7.57	3.35	0.30	0.027	0.006	0.02	0.036
After the filtration process on a catalytic-oxidative sand filter bed (PAXXL1911 coagulation)	7.85	3.40	2.00	0.280	0.015	0.02	0.029
After the filtration process on a dolomite filter bed (PAXXL10 coagulation)	7.75	3.45	0.25	0.017	0.005	0.02	0.020
After the filtration process on a dolomite filter bed (PAXXL1911 coagulation)	7.95	3.50	1.50	0.240	0.015	0.02	0.020
After disinfection (PAXXL10 coagulation)	7.65	3.45	0.24	0.015	0.005	0.02	0.020
After disinfection (PAXXL1911 coagulation)	7.75	3.50	1.10	0.220	0.013	0.02	0.020

**Table 3:** Contents of organic substances, color, and value of Zeta potential and SUVA_254_ indicator in water after individual stages of its treatment.

Sampling Point	TOC mg/L	DOC mg/L	UV_254_ m^−1^	UV_272_ m^−1^	Colour 410 nm	Colour 340 nm	Zeta Potential mV	SUVA_254_ m^2^/g_C_
Mixed water	8.231	7.827	19.338	16.165	40	26	−11.4	2.471
After PAXXL10 coagulation and sedimentation	5.185	5.000	9.800	7.600	7	14	−5.30	1.960
After PAXXL1911 coagulation and sedimentation	5.926	5.650	13.00	10.00	11	16	−8.77	2.300
After the filtration process on a catalytic-oxidative sand filter bed (PAXXL10 coagulation)	4.774	4.700	8.900	6.900	5	11	−5.00	1.840
After the filtration process on a catalytic-oxidative sand filter bed (PAXXL1911 coagulation)	5.550	5.415	12.40	9.500	9	13	−8.77	2.290
After the filtration process on a dolomite filter bed (PAXXL10 coagulation)	4.500	4.400	7.50	5.430	3	7	−4.50	1.700
After the filtration process on a dolomite filter bed (PAXXL1911 coagulation)	5.250	5.100	11.20	8.000	7	10	−7.00	2.190
After disinfection (PAXXL10 coagulation)	4.432	4.356	6.900	4.154	2	6	−3.50	1.580
After disinfection (PAXXL1911 coagulation)	5.145	5.049	10.39	7.380	6	8	−6.00	2.060

**Table 4:** Parameters of linear correlations between the iron (III), turbidity, and the organic matter indices of the water samples after PAXXL1911coagulation.

Linear Correlation Equation	Coefficient of the Pearson Correlation (R)
Fe(III) = 0.0206 UV_254_ − 0.1334 Fe(III) = 0.0523 DOC − 0.1554 Fe(III) = 0.0474TOC − 0.1500 Turbidity = 9.9331Fe(III) + 1.2595 Turbidity = 0.2431UV_254_ − 0.8480	0.9389 0.9225 0.8936 0.9489 0.8526
Turbidity = 0.613DOC − 1.0858 Turbidity = 0.5509TOC − 0.9885 Colour_410_ = 74.105Fe(III) +34.041 Colour_340_ = 4.156TOC − 5.8122 Colour_340_ = 4.4747DOC − 5.6988 Colour_340_ = 1.7273UV_254_ − 3.3383	0.8322 0.7982 0.8834 0.9743 0.9829 0.9802

**Table 5:** Selected properties of the tested coagulants and the degree of polymerization of the coagulants according to the conventional ferronometry [51,52].

Indicator	Type of Coagulant	
	PAXXL10	PAXXL1911
Alkalinity ratio, [OH^−^]/[Al^3+^]	2.10	2.55
Alkalinity, %	70	85
Al^3+^, %	5.0	11.5
Fe_tot_, %	-	0.7
Cl^−^, %	11.5	7.0
Monomeric Al species (Al_a_), %	22.4	14.3
Polymerized Al species (Al_b_), %	29.0	40.0
Colloidal Al species (Al_c_), %	48.6	45.7

**Table 6:** Water sample collection points after subsequent stages of water treatment.

Sample Number No.	Water Sample Collection Points
1	Groundwater
2	Groundwater, following aeration
3	Surface water from the Obrzyca River
4	Surface water, following microfilters
5	Mixed water—groundwater after aeration and surface water from the Obrzyca River after the microstraining process, mixed in a volume ratio of 1:2
6	After PAX XL10 coagulation and sedimentation
7	After PAX XL1911 coagulation and sedimentation
8	After the filtration process on a catalytic-oxidative sand filter bed
9	After the filtration process on a dolomite filter bed
10	After disinfection with chlorine dioxide-water directed to the network

## Sample Availability

Samples of the compounds are not available from the author.
